# Medication and test prescription by nurses: contributions to advanced practice and transformation of care*

**DOI:** 10.1590/1518-8345.2423-3062

**Published:** 2018-10-25

**Authors:** Wezila Gonçalves do Nascimento, Severina Alice da Costa Uchôa, Ardigleusa Alves Coêlho, Francisco de Sales Clementino, Maria Valéria Beserra Cosme, Rayone Bastos Rosa, Isabel Cristina Araújo Brandão, Claudia Santos Martiniano

**Affiliations:** 1 Prefeitura Municipal de Campina Grande, Secretaria Municipal de Saúde, Campina Grande, PB, Brazil.; 2 Universidade Federal do Rio Grande do Norte, Departamento de Saúde Coletiva, Natal, RN, Brazil.; 3 Universidade Estadual da Paraíba, Departamento de Enfermagem, Campina Grande, PB, Brazil.; 4 Universidade Federal de Campina Grande, Unidade Acadêmica de Enfermagem, Campina Grande, PB, Brazil.; 5 Universidade Federal do Rio Grande do Norte, Programa de Pós-graduação em Ciências da Saúde, Natal, RN, Brazil.

**Keywords:** Nurse, Prescription of Medications, Request for Examination, Primary Health Care, Transformation of Care, Advanced Practice Nursing

## Abstract

**Objective:**

To carry out a documentary study on the rules, guidelines, policies and institutional support for the nurse to prescribe medicines and request tests with a view to the advanced practice in the scope of Primary Health Care.

**Methods:**

Documentary research using open-access institutional documents - Federal Nursing Council (COFEN), its regional representations in the respective Brazilian states (COREN) and the Brazilian Nursing Association (ABEN).

**Results:**

Most of the news/notices were issued by the Regional Nursing Councils in the different Federative Units. The argumentation regarding the prescription of medicines and request for tests by nurses is based on three categories: Autonomy and competencies for the prescription of medicines and/or request of tests; Corporate policies that undermine the full exercise of nursing; and Transformation of health and nursing care in Primary Health Care.

**Conclusion:**

The prescriptive practice by nurses integrates health care and has been defended by the institutions that represent the category. It emerges as an important element of advanced practice and in the transformation of care in the context of health teams.

## Introduction

Nursing has been highlighted as the profession with the greatest tendency to develop in the 21st century. In terms of its institution as a profession, it has advanced more in some countries^1^ and less in others, especially in developing countries, such as in Latin America and Brazil^2^.

Nursing has great importance in the process of caring, raising needs and meeting them in the light of the social determinants of the health-disease process. It is important to note that, according to a report from the Pan American Health Organization (PAHO), 60% of the workforce in the health area consists of nursing staff, with about 20 million nurses distributed worldwide, and ¼ of that contingent is in the region of the Americas. However, in Latin America, there are 15 nurses for every 10,000 people, when the expected is at least 23 professionals for this population^2^. This also highlights the shortage of this professional in health systems for political, ideological and cultural issues.

Besides the shortage of these professionals, they also have not developed the provision of care in a holistic and comprehensive way, given that corporate policies tend to inhibit or hinder the exercise of nursing. Among the most emblematic issues, there is the medication and test prescription, an important and common practice in Primary Health Care (PHC), which the medical category has made efforts to discourage or inhibit. However, it is one of nurses’ attributions, especially in view of those common conditions or needs of the community^3^.

There are few studies in the literature that include medication prescription. In the United Kingdom^4^, United States^5^ and Canada^6^ it is a duty of this professional to fully assist the users, which includes the prescriptive practice. However, in Brazil, this is still undefined, and there are doubts and controversies about the ethical, legal and institutional bases for the practice of prescribing medicines and requesting tests, when performed by nurses^7^. In terms of knowledge gaps, there are few studies analyzing medication prescription by nurses in the context of advanced practice^8^, although this is an important issue to be addressed in order to train and motivate the nurses on this issue in PHC, in Brazil.

PAHO has commissioned an inquiry into the advanced nursing practice and has included the prescription of tests and medications^8^. The transformation of nursing care is a *sine qua non* for advancing the profession and improving the quality of health services in the country.

Nursing, as a category, in Brazil is represented by two main institutions, namely the Federal Nursing Council (COFEN) and its regionalized bodies; the Regional Nursing Councils (COREN) and the Brazilian Nursing Association (ABEN). In this sense, the research question is how the nursing institutions are positioning themselves on the medication prescription by nurses in the scenario of Primary Health Care? The present article had the objective of carrying out a documentary study on the rules, guidelines, policies and institutional support for the nurse to prescribe medicines and request tests, with a view to the advanced practice in Primary Health Care.

## Method

This study was the result of qualitative, exploratory and documentary research. The documents were open-access, official and institutional, that is, originating from COFEN, its regional representations in the respective Brazilian states - COREN, as well as ABEN documents.

The nursing institutions present themselves as spaces that represent the thinking of the professional category throughout history. The COFEN/COREN system gives recognition to Nursing as a health profession, allowing the extension of its autonomy and the application of scientific knowledge for the professional exercise. ABEN’s mission is the social, political and scientific development of the profession. Created in 1926, it is an entity that has a great national expression in relation to the Nursing Curricular Guidelines. Its performance transcends the disciplinary and/or regulatory nature; it is ahead of the movements that aim at the transformation and empowerment of Brazilian nursing^9^.

The documents were selected by quality criteria^10^ that encompass authenticity (primary document), credibility (documents without errors or distortions), representative (typical of the institution) and meaning (clear and comprehensive).

Intentional sampling was used to build a corpus that is represented here by the contextualization of the medication prescription by nurses in the PHC in Brazil, in which the two organizations representing the nursing category, COFEN and ABEN, have preponderant roles on professional practices. The documents were selected by convenience, seeking the intertextuality between the national and regional/state levels that could point out the different connections or confluent institutional positions (councils and association) about the regulation and management of conflicts before other prescribers and the society. We analyzed 39 technical news/notices and 19 technical opinions that sought social and scientific legitimation; and three features of ABEN’s Journal from 2003 to 2016. The delimitation of this time period is justified by the value character that the PHC starts to have in the Brazilian Health System, one of the practice scenarios of the prescribing nurse.

The COFEN documents were collected on its official website (http://www.cofen.gov.br/) and its state correspondents in order to collect local or regional data. The search was performed using the descriptor “medication prescription”. The study included documents that dealt with medication prescription by nurses from 2003 to 2016. Duplicate documents found simultaneously in COFEN and COREN were excluded, as well as news from other entities published on the website without the formal position of COFEN/COREN.

The collection was also carried out on the ABEN’s Journal website (http://www.abennacional.org.br/home/apresentacaojornal.htm). Considering that the website does not have a search tool, we read each issue of the journal was read, identifying those that dealt with the theme of medication prescription. Collection was held in the period from January to March 2017 and were carried out by two pairs of researchers, and the documents that reached consensus were selected.

The texts were identified by a previously validated script by means of the Delphi Technique, being sent to five experts in the theme. The script included questions on whether or not having medications prescribed by nurses.

After collection, the data were organized according to the origin and type of the document; then, they were systematized into analysis categories.

For the analysis, we followed the methodological guidelines of content analysis, thematic modality: pre-exploration of the material or floating readings of the *corpus*; selection of units of analysis (or units of meanings); categorization process and subcategorization^11^. The units of meanings were identified in the text by a reference code according to the type of documents: News/Notices (NN), Opinion (O) and Journal (J), followed by the order number in which they were organized.

Since this is a research with public and open-access data, it required no submission to the Research Ethics Committee.

## Results

**Figura 1:**
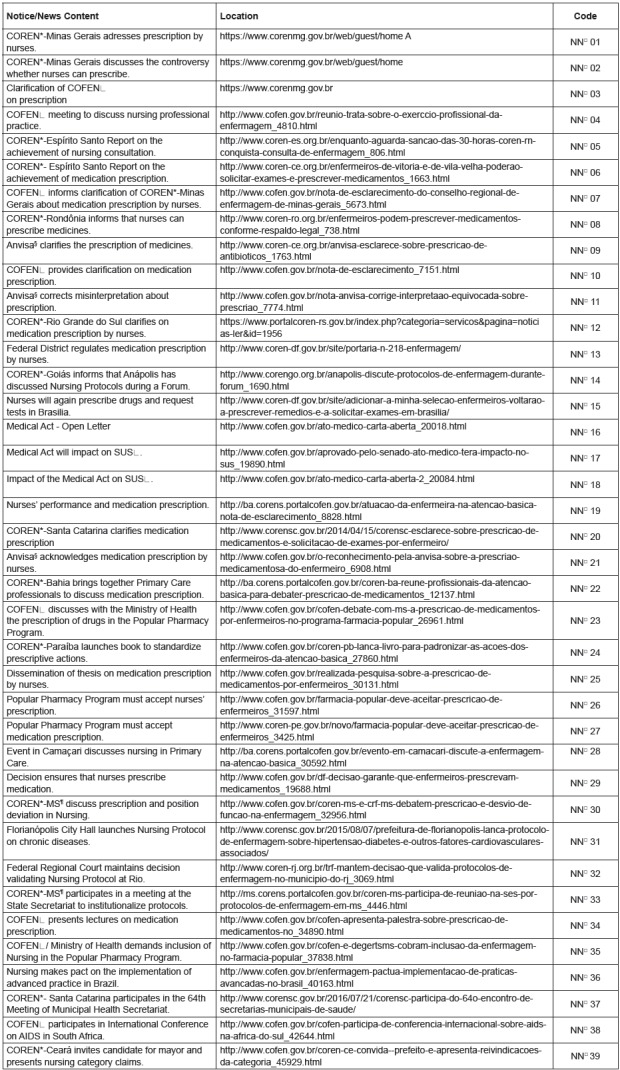
News/Notices from CORENS*/COFEN^ǂ^ related to the issue of medication prescription by nurses (2007-2016)

**Figure 2:**
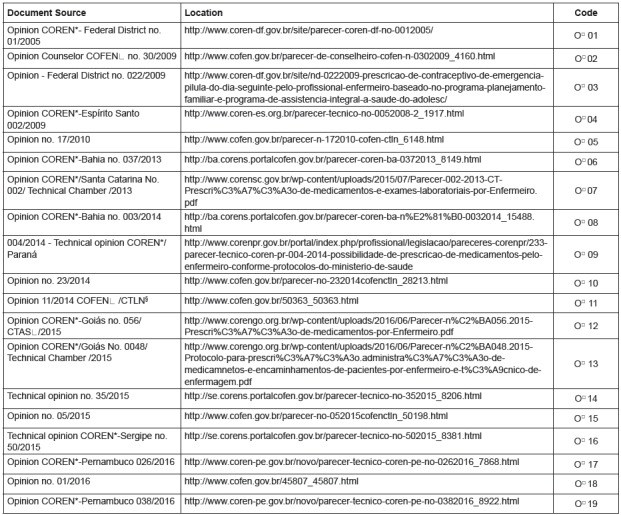
CORENS*/COFEN^ǂ^ opinions involving the issue of medication prescription by nurses (2005-2017).

Figures 1 and 2 characterizes the 62 documents, considering the saturation criterion and taking into account the document type, date, location and sequential coding. Figure 1 lists the main Brazilian news reports on the nurses’ practice, especially regarding the prescription of medications and the request for tests. It can be observed, according to the Figure, that most of the news or notices were transmitted by the Regional Nursing Councils in the different Federation Units; the news can be accessed in full through the link contained in the said figure. 

*COREN - Regional Nursing Council; ϯNN - Notices and News; ǂCOFEN - Federal Nursing Council; §Anvisa - National Health Surveillance Agency; ǁSUS - Unified Health System; ¶MS - Ministry of Health

*COREN - Regional Nursing Council; ϯO - Opinion; ǂCOFEN - Federal Nursing Council; §CTLN - Technical Chamber of Legislation and Norms; ǁCTAS - Technical Chamber of Health Care

Figure 2 lists the main opinions issued by COFEN or COREN on the medication prescription and request of tests, evidencing that the nursing category started to worry about this theme more noticeably from 2005, according to online documents accessed.

**Figure 3:**
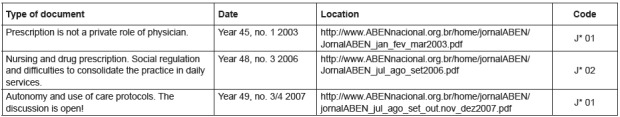
ABEN^ϯ^ Journal articles involving the issue of medication prescription by nurses (2003-2016)

Figure 3 shows articles released by ABEN about its position regarding medication prescription by nurses. 

*J - Journal; ϯABEN - Brazilian Nursing Association

Next, the qualitative aspects of each document selected for the study were listed. Considering the theoretical framework defined for the research, three central categories emerged: Autonomy and competencies for the prescription of medicines and/or request for tests; Corporate policies that undermine the full exercise of nursing; and Transformation of health and nursing care in Primary Health Care.

The first category observed in the analyzed documents was the affirmation of nurses’ autonomy and competence for prescribing and requesting tests, which was addressed both to society and to the nursing category itself, based on the Professional Exercise Law No. 7,498/86, in the institution of Public Health Programs in routines established in the Protocols or Primary Care Booklets. This statement was verified in technical notices/news and COFEN/CORENS opinions. *The Regional Nursing Council of Bahia (COREN-BA) hereby reaffirms to society and nursing professionals that nurses have the autonomy to prescribe medications and to request tests in the scope of public health programs through the Manuals of the Ministry of Health and routines approved by health institutions, according to protocols.* (NN 09).

The debate on the autonomy of this profession was also raised by ABEN in 2007 when it published a report on autonomy and use of care protocols, which dealt with the transcription/prescription of medicines. Among the advantages of prescriptive practice is the technical autonomy, as reported in the article: *I perform the preventive examination for cancer, the clinical examination of the breasts and I find that the woman has a candidiasis, for example. How can I think of this woman’s well-being if I do not have the support to prescribe a medication for this type of problem? The poor woman has to return the other day before dawn to schedule a consultation with the doctor. [...] Dealing with women with this holistic perspective of care implies more technical autonomy to provide care. It is necessary something that at that moment makes a difference for this woman; it needs to be resolute; returning the other day for a medical consultation is very difficult to reach.* (J 03).

The second category perceived in some documents was the corporate policy that impairs the full exercise of nursing. *In November 2006, the Federal Medical Council, the Medical Association and the National Federation of Physicians filed a writ of mandamus against the Federal Government requesting an injunction, which was filed at the 4th Federal Court of the Judicial Branch of Brasília under No. 2006.34.0034.729-1, with a view to declaring the nullity of Administrative Rule MS 648/GM/2006 and its annex (that is its object). At the first moment, the Federal Judge of that Federal Court rejected the request for a preliminary injunction, and the Federal Medical Council filed the Bill of Review no. 2007.01.00.000126-2 for the Federal Regional Court of the 1st Region, with no success.* (NN 10). *At the beginning of the year [2010], the Federal Justice Court condemned the Regional Council of Medicine of Espírito Santo (CRM-ES) to indemnify for moral damages a nurse from the Family Health Program (PSF) in Vitória. During a home visit, she had requested preventive examinations and prescribed a medication to a resident of the city assisted by the program. Upon learning of the case, the CRM filed suit against the professional, claiming illegal medical practice. However, the nurse was cleared by the Federal Court itself and filed suit against the council for feeling harmed. (NN 06). The possibility of conducting nursing consultation and prescribing medications established in public health programs and routinely approved by the health institution are competencies provided in art. 11, of Law No. 7,498/87, and no judicial decision has declared suspended or non-enforceable such provisions of the law that regulates the nursing professional practice. We sincerely hope that the regional councils of medicine will restore the truth and recognize the legal competencies attributed to the esteemed nursing class.* (NN 03).

In response to the Federal Medicine Council, nurses’ representations answered questions on the prescriptive practice, resorting to the norms of the Ministry of Health, through the National Agency of Sanitary Surveillance (Anvisa), in which the Resolution of the Anvisa Collegiate Board (RDC) recommends, in the context of the Family Health Program (FHP), prescription by any qualified professional and does not translate the exclusivity to the medical practice. The reference to the prescription of antibiotics by the Ministry of Health is to clarify this issue, as follows: *When asked whether the nurse could continue to prescribe antibiotics in the PSF, the MS [Ministry of Health] coordinator replied that in art. 3 of the RDC [Resolution of the Anvisa Collegiate Board], it is stated that the prescriptions can only be dispensed when presented in a legible manner, with no erasures, by duly qualified professionals, which does not limit the prescription of antimicrobials to the medical professional in the Family Health Program. He emphasized: the RDC [Resolution of the Anvisa Collegiate Board] did not withdraw the requirements contained in law 5991/73 from any professional qualified for the prescription of antimicrobial drugs* (NN 09).

The advocacy of the attribution of prescribing and requesting tests was highlighted in legal actions. The confrontation with the medical category was also incorporated by ABEN. In 2003, the journal published an article stating that prescription is not a private act of the physicians. The article denounced attacks from representative entities of the medical category through to nursing prescription. It also addressed the national movement articulated by the medical category towards restricting medication prescription to physicians. The author of the article pointed out that [...] *by attacking the freedom of nursing professional practice, with its own legal regulation, the movement articulated by medical entities also violates fundamental rights set in the Constitutional Charter.* (J 01).

The third category highlighted in the present research was the transformation of health and nursing care in the scope of Primary Health Care. From this category, five subcategories emerged. The first subcategory was prescription as a factor of accessibility, safety and focus on users.

In response to lawsuits motivated by medication prescription by nurses in PHC, the COREN/COFEN drew attention to the fact that it is a safe practice that contributes to the accessibility of medicines and focuses on users of public health services. *In the court decision, it was understood that the nurses’ performance does not harm public health, but rather contributes to a more immediate and effective provision of medical services, representing greater accessibility to public health services, in which the patient is privileged.* (NN 03).

The adoption of accessibility discourse has justified the search for formalization of prescription in local health services through the establishment of local protocols. *In order to increase the quality of access to health services in Florian*ó*polis and to support the performance of the nursing professionals, the nursing team of the Florianópolis Municipal Health Department has collectively developed the Nursing Protocol on Hypertension, Diabetes and other associated cardiovascular factors.* (NN 32).

In other situations, the spaces left by other professionals have been occupied by the nurse in the perspective of user care resolution. This was the justification used by the Municipal Health Department of São Paulo when asking the COREN-SP opinion on the prescription of emergency contraceptives. *The* *Prescription of Emergency Contraceptives (morning-after pill) by the nurse based on the Family Planning Program and the Comprehensive Care Program for Adolescent Health due to the increased demand and deficit of gynecologists in the Basic Health Unit. Also, since it is an emergency contraceptive, there is no possibility of waiting for a consultation.* (O 03).

The second subcategory was medication prescription by nurses as a stimulus to teamwork and a change of Primary Health Care. In this sense, the analyzed documents revealed that the advocacy of COFEN/COREN to medication prescription by nurses is based on the possibility of consolidating the change of the primary care model focused on the team work with respect to professional competences: *Nurses play an important role in prevention and health promotion programs, which is precisely the model we want to see fully operating in the country. For this purpose, the work of professional teams is crucial, respecting their technical and legal skills. (NN 06). For the Counselor [name of the Counselor], the attacks on the nurses’ competences have occurred in several localities of the country. This is because, according to the counselor, a paradigm shift is observed within the whole health network with regard to primary care.* (NN 03).

ABEN’s firm belief in the possibility of changing this model was verified through an article entitled “ Nursing Prescription of Medications: Social Regulation and Difficulties for the Consolidation of this Practice in the Daily Routine of Health Services”, which discussed the advances of prescription within public health policies since the creation of the Ministry of Health with programs, actions and articulations of the Unified Health System (SUS). *The nursing practice in Primary Health Care has been already strongly regulated and organized by protocols, agreed upon for teamwork and, in this respect, in the daily routine of services, the accomplishment of these duties of the nurse has been crucial to the development of health actions and programs, [...] directly influencing the quality of life of individuals, families and communities.* (J 02).

The third subcategory was the medication prescription by nurses as a way to reduce costs with human resources in the health area, since the COFEN/CORENS has a mobilizing discourse on the prescription of medicines by nurses as a form of reducing costs in the sector. *For [name], an inspector of the Regional Council of Nursing, doctors often do not account for the demand in public health and, therefore, it is important to assign to the nurse responsibilities for which they are competent* (NN 15).

However, it is necessary to be aware of the implications of this attribution. *A counselor [name] discussed with the nurses on the challenges faced by them in primary care, such as work overload, the inclusion of medication prescription in the work process without a corresponding wage increase, the persistence of conflicts in the work with other professionals and with physicians who do not recognize the nurse’s technical capacity.* (NN 37).

The fourth subcategory is the prescription and the technical competence provided by the training, which is affirmed by the representative entities as an inherent condition for the nurse’s training. *Regarding the request for routine exams, nurses are allowed to request them, when they have a legal and technical competence to carry out the reading of the requested test with efficiency. (NN 07). The nurse is able to prescribe drugs in family health units where all medicines are provided free of charge and cannot be sold* (*O* 03). *In the nurse’s academic training, he/she attends the PHARMACOLOGY discipline, the same as that of the medical professional, so he/she is aware of pharmaceutical drugs, posology, interactions, side effects, and other information necessary for the prescription thereof.* (*O* 01).

Finally, the fifth subcategory evidenced that the prescription of medicines should be part of the Systematization of Nursing Care (SAE), once it was noticed in the COFEN/CORENS documents the insertion of the prescription in SAE as a way of evaluating the decisions in knowledge, nursing competencies and resolutions. *Nursing procedures should always be supported by scientific rationale and should be carried out through the effective elaboration of the Systematization of Nursing Care and the Nursing Process, provided for in Cofen Resolution 358/2009. (O 16). We suggest that, in time, study groups on SAE [Systematization of Nursing Care] are structured aiming at training the nursing team to use the Nursing Process as a tool to operationalize the protocols of the Ministry of Health.* (O 06).

## Discussion

The objective of the present research was to conduct a case study on the regulations, guidelines, policies and institutional support of nurses for the prescription of medications and request for tests with a view to the advanced practice of nurses in Primary Health Care.

Nurses’ autonomy for the prescription of drugs has been widely reaffirmed by the nursing class entities and is in line with the expansion of the prescriptive autonomy affirmed in Primary Care protocols adopted by the Ministry of Health. Brazil has been following the trend of several countries, such as the United Kingdom, where nurses have the greatest prescriptive power in the world, as they develop this practice independently, for all health conditions, including controlled medicines, within the scope of their clinical competence^4^. The authority to prescribe medications contributes to the expansion of nurse’s autonomy and is among the characteristics of advanced practice nurses^12^.

The movement against the prescriptive action of nurses has been articulated by the medical category since 2006, when the first Opinion of the National Policy for Primary Care was published, and took on greater importance during the whole process of the regulation of the medical profession, completed in 2013. The law comprised decisive vetoes in maintaining nurses’ prescriptive action. Physicians’ dispute of prescription as a privative role is based on the defense of the monopoly and of the privileges of their category. Their struggle is on the labor field, but the results are economic, reflecting the interest of a competitive society, a globalized world and a neoliberal economy^13^.

In the international context, the prescription of medicines by nurses is considered an advanced practice in nursing and is part of the innovations of the category. In Sweden, government agencies made a positive assessment of the nurse’s prescriptive action, as there was improved communication and access to services. Also in the United Kingdom, this practice has made access easier and in South Africa there has been an improvement in care and a great benefit to the community, especially in rural areas^14^.

In conformation of a new model of care guided by the principles and guidelines of the SUS, which has Family Health as a priority strategy for Primary Health Care, the nurse assumes a prominent role, since they receive attributions that contribute to the universal access and coverage of health services^15^. Throughout the world, team care has been considered as paramount for primary quality care^16^.

Since 2013, PAHO has also signaled the strengthening of health systems in order to progressively increase the quality and provision of care aimed at meeting the basic needs of the human being. PAHO’s intention is to give autonomy and support to collaborative multi-professional PHC teams based on established models of care and to maximize the scope of practice of each profession by its own competence, including advanced practice nurses^8^.

One of the major concerns of health systems today is cost reduction. One of the strategies to achieve this goal is redesigning the functions among health professionals. In this sense, the nurse has been called to expand their functions. Thus, it is believed that, when nurses become prescribers, they enhance patients’ access to medications and increase the availability of prescribing professionals in health services^17^. However, it is advocated that the prescription of medicines by nurses, by increasing the access of drugs to users, is not on the order of normative access to the rule of law, but the aspect of the comprehensiveness of care that is a prerogative to that rule of law. It cannot be conceived that the user does not have full access, when there is the prerogative of the right instituted and the ministerial normalization.

A study carried out with nurses of the Family Health strategy in Campina Grande-PB on prescription training revealed that only some of them felt prepared and pointed out the Pharmacology discipline as one that could offer support to this practice^18^. Therefore, even though the COFEN/COREN states the nurses’ technical competence for prescribing, the nurses themselves do not feel prepared for such practice. This situation does not seem to be isolated, since in Brazil there is no requirement for specific training of nurses to prescribe medications, which can lead to professionals who are not fully prepared for this assignment. It is believed that strategies such as Permanent Education; the establishment of reference matrix teams for the support of prescribing nurses; and resources of the Telehealth Program constitute tools to support the prescriptive practice of nurses, solidifying it.

It should be emphasized that the nursing process is based on the Systematization of Nursing Care (SAE) and that any nursing action should be the result of this process. It should be noted that the protocols published by the Ministry of Health do not consider SAE because they are protocols for health professionals, and not specifically for nurses. However, it was verified in the state of Paraíba the initiative to implement the SAE within the Nurse Protocol in the Family Health Strategy of the State of Paraíba as an instrument guiding the evidence-based practice^19^.

The study advances in systematizing and demonstrating the convincing argumentation of the representative entities diluted in many disputes, but directed to the category, since the discussion of medication prescription by nurses should be aligned with its primary purpose that is the user and comprehensive care, which is necessary and important for PHC. We suggest other studies that, besides the thematic analysis carried out, deepen the analysis of the argumentative repertoire as analysis of rhetoric.

The present study has as limitations the use of documents available online. It would be interesting to search *in loco* other sources of data to triangulate with the results of the research. It is important to develop investigations that could map the nurse’s autonomy to the prescribe medicines and request tests according to Brazilian regions, which would give a national idea of how far the regions have advanced in the question and to the advanced nursing practice.

## Conclusion

In Brazil, when considering the normative, legal and ethical conjuncture that instruct the profession, the prescription of medications and request for tests have been affirmed. However, in the political and social aspects, the health care performed by nurses with respect to their prescriptive practice still demands legitimacy, which has been defended by the entities that represent the category.

The expansion of the role of nurses has resulted in a change in the scope of practices traditionally delegated to physicians only, as in the case of drug prescription. The advocacy of representative nursing entities refers to a defense of the autonomy of the profession and should not be interpreted as a threat to other categories.

Because of the complexity of this practice and the potential to contribute to comprehensive care to the user, a principle of rule of law, the prescription of medicines by nurses in PHC emerges as an important element of advanced practice and in the transformation of care in the health context.
